# The Fire That Does Not Go Out: The Neglected Costs of Gas Flaring in Nigeria

**DOI:** 10.3390/ijerph23040430

**Published:** 2026-03-30

**Authors:** Omoniyi Babatunde Alimi, John Gibson

**Affiliations:** 1 Health Global Practice, World Bank Group, 1818 H Street NW, Washington, DC 20433, USA; 2 Department of Economics, University of Waikato, Hamilton 3240, New Zealand; john.gibson@waikato.ac.nz

**Keywords:** gas flaring, infant and child health, stunting, disease incidence, oil and gas production

## Abstract

**Highlights:**

**Public health relevance—How does this work relate to public health issues?**

- The paper examines gas flaring as an environmental public health risk in Nigeria’s Niger Delta, where many communities live close to flare sites.
- It focuses on child health, assessing whether flaring exposure is associated with illness, poor nutrition, and mortality among children under 5 years of age.

**Public health significance—Why is this work of significance to public health?**

- The study provides one of the first large-scale analyses of gas flaring and child health in Nigeria, where evidence has previously been limited.
- It generates new evidence on the wider health costs of routine gas flaring in a major oil-producing region.

**Public health implications—What are the key implications or messages for practitioners, policy makers, and/or researchers in public health?**

- The findings suggest that gas flaring should be treated not only as an environmental issue but also as a child health concern.
- The study also shows how satellite data can help researchers assess environmental health risks in settings with limited monitoring data.

**Abstract:**

Gas flaring, the burning off gas coming out of oil wells is a common practice in oil-producing developing countries. This practice is harmful to human health, especially because of pollutants. This research focuses on Nigeria, where over 10 percent of all gas produced is flared and about 2 million people are estimated to live within 4 kilometres of a flare site. This paper uses child health data from Demographic Health Surveys and satellite-detected data on gas flaring to examine associations between flaring exposure and child morbidity, nutritional outcomes, and mortality among children under 5 years of age. The findings show a positive association between flaring and the incidence of respiratory diseases and fever among children under 5 years of age but no robust association with mortality. The study contributes to the literature measuring the wider cost to society of oil and gas production and adds to the body of work using satellite data to understand well-being in places where conventional data sources are unavailable.

## 1. Introduction

Routine natural gas flaring, the combustion of the gas associated with crude oil extraction, has been a feature of Nigeria’s oil industry ever since production began in the Niger Delta region in 1956. While routine burning of gas represents a potential income loss for a poor developing country, it also likely imposes wider human health costs. Combustion of natural gas is a prominent source of air pollutants such as carbon monoxide (CO), carbon dioxide (CO_2_), volatile organic compound (VOC), sulphur dioxide (SO_2_), polycyclic aromatic hydrocarbon (PAH), nitrogen oxides (NO_X_), and particulate matter (PMx) in the form of soot or black carbon, which can travel over long distances due to the height at which flaring takes place [1,2]. The small but growing literature has examined health impacts of gas flaring, predominantly in the context of unconventional oil and gas production (fracking) in the United States. Studies from North Dakota, Texas, and Pennsylvania have found that proximity to flaring is associated with increased respiratory-related hospital visits [3], higher odds of preterm birth [4], lower birth weight [5], and increased respiratory disease hospitalisations, particularly among children [6]. However, these studies are from developed-country settings where fracking introduces additional exposure channels—notably groundwater contamination from well injection [7,8,9]—that may be less relevant for conventional oil and gas production. The extent to which these findings are transferable to the context of routine gas flaring from conventional production in developing countries remains unclear.

In Nigeria, these risks are especially salient because oil and gas infrastructure is located unusually close to where people live, with an estimated two million people residing within 4 kilometres of a gas flare [10] and also in a broader environmental health context in which oil-related pollution in the Niger Delta has already been linked to a high burden of adverse health and mortality outcomes and community distress [11,12]. This study examines the often-neglected wider cost of oil production in Nigeria by focusing on the human health impacts among children under 5 years of age in the oil-producing Niger Delta region of Nigeria. It links the satellite data on gas flaring to the georeferenced child health outcomes data from Demographic Health Surveys and examines the association between flaring and child health (incidence of diseases), nutritional characteristics (short-term and long-term measure of child nutritional and health status), as well as death among children. We find a positive association between gas flaring and the incidence of disease, specifically, cough, respiratory illness, and fever, and positive associations between flaring and the probability of a child being either wasted or underweight but no association with deaths.

There are several reasons to examine the wider health costs of flaring in the Nigerian context. First, most of the existing evidence on the health impacts of flaring are from developed countries in the context of unconventional gas production (fracking), whereas gas flaring in Nigeria is associated with conventional oil production. This distinction matters, as the process of fracking has been associated with other forms of environmental mechanisms that may affect human health, such as ground water pollution from well injection, which may not solely be due to gas flaring [7,8,9,13]. Notable exceptions like [14], which examine both conventional and unconventional natural gas development in Texas, find that conventional drilling has similar, if not potentially larger, risk for health (paediatric asthma hospitalisations) compared with unconventional drilling. Second, weak regulatory and policy settings in Nigeria make it easier for companies to flare the gas rather than find alternative uses. For example, it was not until recently that the 2018 Flare Gas (Prevention of Waste and Pollution) Regulations increased the penalty for flaring by around 80-fold, from US$0.024 (10 Naira) per thousand standard cubic feet (SCF) to US$2 per thousand SCF for companies that produce at least 10,000 barrels of oil per day [15]. Third, the examination of the health impact of both onshore and offshore flaring also has important policy implications in Nigeria, where there is a long-standing debate about the distribution of the gains from oil exploration to areas bearing the costs and between the various levels of government administrations and oil-producing communities [16]. Fourth, the child health focus of this study is also well-motivated in Nigeria, where prior analysis of preschool children has shown that symptoms of disease incidence of acute respiratory infection are strongly associated with environmental and household risk factors and vary substantially across settings [17].

Our study thus makes three important contributions. First, it provides the first comprehensive examination of gas flaring impacts on child health, in the country that contributes the most (by volume) to gas flaring in Africa and the seventh most globally. Moreover, most existing evidence is from richer countries (particularly US), where confounding due to the endogenous avoidance behaviour potentially complicates the analyses. In Nigeria, the avoidance behaviour may be more limited in this setting, especially due to land market rigidity in the densely populated Niger Delta area that stems from concerns over the distribution of oil wealth and the huge ethnic divide in this area. The Niger Delta is home to over 40 different ethnic groups, and the location of oil infrastructures determines the benefits enjoyed by the host community including patronage, loyalties, jobs, contracts, scholarships, social facilities, and compensation [18]. Rent from oil and gas companies to communities and the ethnic divide promotes a strong attachment to land in this area. The second contribution of our study is the consideration of offshore gas flaring. Most evidence on health impacts of flaring is from land-based settings, reflecting a preponderance of studies on fracking. While a large portion of literature considers environmental impacts of offshore oil and gas production [19], little is known about impacts of offshore gas flaring on human health. Yet, half of all of Nigeria’s flaring is offshore, with three quarters of these flaring locations located within 60 km of areas where people live. The evidence has shown that the type of pollutants generated by flaring can affect air quality up to 108 miles (174 km) away [20], so it is likely that offshore flaring has an impact on coastal communities. Finally, our study demonstrates the value that can be added to the georeferenced survey data by linking to satellite observations of environmental phenomena, especially for countries that lack the wherewithal for conventional monitoring [21,22].

Nigeria has the largest proven gas reserves in Africa (about 201 trillion cubic feet), or about 2.7 percent of global proven reserves of gas [23]. The latest statistics from the Nigerian Department of Petroleum Resources (DPR) indicated flaring volumes of 321 million SCF for 2018—around 11 percent of total gas produced in the country for that year [24]. Why does a low-income, energy-insecure country like Nigeria routinely burn a valuable source of energy and a potential income earner? First, there might be technical reasons for flaring, such as releasing pressure in oil wells, but a focus on production of crude oil for refining or export makes gas an unwanted by-product. Gas capture and processing require different infrastructure and investments that oil companies, typically set up to extract and transport crude oil, may be unwilling to make. Second, the weak regulatory and policy settings make flaring an attractive choice. Prior to the enactment of the July 2018 Flare Gas Regulations, based on the flaring volume, gas producing companies would only have been liable for a fine of around US$8 m (with 100% compliance with the regulations) but with the enactment of the law, the fine amount will have been around $642 m with 100% compliance based on official 2018 flare volumes. Yet, ref. [25] estimated a direct revenue loss of US$761 m from flaring in 2018 (this excludes lost value from the derivatives that can be produced if the gas was further processed), putting into perspective the wasteful impact of flaring on the Nigerian economy. To give another comparison, the value placed on gas burnt at the well is around 4 percent of annual fiscal revenue and 13 percent of the fiscal deficit in the 2018 national budget. Moreover, the situation was even worse in the past, with about half of all gas produced being flared in 2002. The ongoing use of gas flaring reflects limited progress in commercialising the gas (including gas-using industrialisation) and in technological improvements. Overall, gas flaring represents a great revenue loss in a country with a significant income shortfall.

Despite the huge economic potential of reducing gas flaring in Nigeria and the potential health impacts, to date, there are only a few studies that have examined the health impact, and they typically compare health outcomes in a few communities with and without flaring sites [26,27]. A survey of attitudes in oil-producing communities found residents perceive gas flaring as hazardous but feel resigned to it [28]. Small case studies have linked residence in gas-flaring communities with hypertension [29] and higher frequency of respiratory complaints [26], while the analysis of soil sediments has detected polycyclic aromatic hydrocarbons near oil and gas producing areas [30]. No large-scale analysis has examined the relationship between gas flaring and child health in Nigeria despite over 6 decades of routine flaring.

Yet, the need for evidence from Nigeria is particularly strong. Public health research in Nigeria already shows that environmentally mediated child health risks are substantial. Using the Nigerian data on preschool children, ref. [17] documents the importance of environmental risk factors for acute respiratory infection symptoms, while [31] shows that acute respiratory infection, diarrhoea, and stunting exhibit overlapping spatial risk patterns among Nigerian children under 5 years of age. While these studies do not identify gas flaring as the causal exposure of interest, they underscore the public health relevance of the specific child outcomes examined here.

This paucity of evidence on health impacts of flaring is not surprising because identifying these requires detailed data on air quality, flaring volumes, and health records, which are often unavailable in this setting, and prevents the use of more sophisticated causal inference techniques to establish a causal link between flaring and child health outcomes, unlike in other developed settings [3,6,32]. To bridge this gap, we rely on satellite-detected gas flaring locations (and estimates of gas flaring volumes) and examine the association between gas flaring and disease incidence, child anthropometric outcomes, and death among children under 5 years of age in the oil-producing Niger Delta region of Nigeria. While our analysis is not causal, we provide some initial evidence on the often-neglected health costs of oil and gas production in Nigeria, and our technique could be extended to other oil-producing developing countries that practice routine gas flaring, but where the unavailability of data has prevented the examination of the health costs.

The rest of the paper proceeds as follows: Section 2 describes the data and our method of linking satellite-detected gas flaring locations and estimates of gas flared volumes with human health data from the DHS; Section 3 contains our results; and Section 4 concludes the work.

## 2. Materials and Methods

### 2.1. Data Sources

We use the data from two sources. The first is satellite observation on gas flaring locations and estimates of flare volumes. These come from the Visible Infrared Imaging Radiometer Suite (VIIRS) onboard the Suomi satellite. Launched in 2012, the VIIRS sensors can detect heat emitted by gas flares through the collection of shortwave and near-infrared data at night, recording peak radiant emissions from flares. A methodology for estimating the volume of gas flared at each site, with the accuracy of the flared gas volume estimates rated at ±9.5%, was presented by [33]. These data and methods have been used to identify global flaring locations and to estimate global gas flaring volumes [34]. A publicly available website, www.gasflaretracker.ng (accessed on 1 February 2021), has the geographic coordinates of each flaring point in Nigeria, as well as monthly estimates of the flare volumes from each location. While the satellite data from sources like VIIRS can provide the data on flaring in Nigeria, these data are far from perfect. The VIIRS data has been shown to miss small flares which may be captured by other satellite sensors with finer resolution, such as the Landsat OLI sensor (Landsat’s relatively low temporal frequency limits its usefulness for stand-alone monitoring of gas flares, especially for short-term or rapidly changing activity) [35]. The evidence from settings where the VIIRS data on flaring has been compared to satellites with finer spatial resolution has shown the VIIRS data to be representative and the spatial resolution of VIIRS makes it adequate for monitoring large gas flares typical of conventional oil production compared to the smaller flares associated with unconventional production [36]. Flaring in Nigeria stems from conventional oil production, making VIIRS a good fit for the flare data in our context.

Our child health data come from the 2013 and 2018 Nigerian Demographic Health Surveys (DHSs). These nationally representative cross-sectional surveys have demographic and health details for women aged 15–49 and for children aged 0–5. A stratified, two-stage cluster design is used, with the geo-coordinates of each cluster reported with some displacement for confidentiality reasons (the displacement is up to 2 km for urban clusters and 5 km for rural clusters, with 1 percent of rural clusters displaced up to 10 km). The DHS collects information on women’s birth histories and child health characteristics, including self-reported measures of cough and other respiratory symptoms, as well as fever. Anthropometric measurements are made for children under 5 years of age. An important consideration is the quality of the DHS health data used in this analysis. The child morbidity measures (cough, respiratory symptoms, fever, and diarrhoea) are based on maternal recall over the 2 weeks preceding the interview, which may introduce recall bias and reporting errors [37,38]. Mothers may vary in their ability to recognise and recall symptoms, and reporting may be influenced by access to health services and cultural norms around illness. However, validation studies have generally found maternal reports of childhood illnesses, like those in the DHS, to be reasonable proxies for clinical morbidity in low- and middle-income settings, particularly for acute symptoms such as fever and cough [39]. Moreover, the anthropometric measurements (height and weight) used to construct z-scores for stunting, wasting, and underweight are objectively measured by trained DHS field teams following standardised protocols, and studies examining DHS anthropometric data quality have found that prevalence estimates are robust to data quality correction [40]. For mortality, the DHS birth history data are widely used as a primary source of child mortality estimates in the sub-Saharan Africa, and recent analyses of 204 DHSs suggest that common data issues such as age heaping introduce only modest bias in infant mortality estimates [41]. On balance, while the DHS data have known limitations, they represent the best available source for examining child health outcomes at scale in Nigeria and have been used extensively in the public health literature for this purpose. We focus on all DHS clusters in the oil-producing Niger Delta region and in Cross River State. Although Cross River was delisted as an oil-producing state in 2009, it is in close-proximity to current offshore oil and gas wells off the coast of Nigeria. Appendix A Table A1 provides the summary table of the datasets used in the study.

### 2.2. Method

We use a market potential approach to link gas flaring locations to the risk of exposure to gas flaring for each DHS cluster. Specifically, we calculate the inverse distance weighted average flare volume from each flare site for each DHS cluster:

(1) $${RF}_{ct}=\;\sum_{f=1}^{F}\left(d_{f}^{-1}\right)*{FV}_{f}$$

where $d_{f}^{-1}$ is the inverse of the distance (we use the Stata 16 routine *geonear* to calculate the Euclidean distance between each DHS cluster and each flaring location) between the cluster, c, and flaring location, f; and ${FV}_{f}$ is the gas flare volume in the flaring site, f. This inverse-distance weighted flaring volume for each cluster ${(RF}_{ct})$ is our key variable of interest. There are some advantages to taking this market potential approach. First, the DHS reports the geo-coordinates of each primary sampling unit (cluster) with some displacement and, given the uncertainty around the exact location of the cluster, the market potential which takes into account flaring from all flare sites seems to be very appropriate compared to other approaches, such as those that might take into account flaring from the nearest site to a DHS cluster. Second, this approach does not treat each flaring site as equal but takes into account the volume of gas flared in each location, as well as the fact that flare sites closer to DHS clusters should have more impact on health outcomes than flare sites farther way. Third, this approach allows us to take into account the potential effect of offshore flaring, treating it in an equivalent way to onshore flaring. If, instead, we used flaring averages for the local administrative areas where the DHS cluster was located, the offshore sites would be missed. About half of all of Nigeria’s flaring happens offshore, and three quarters of these flaring locations are located within 60 km of areas where people live, which is well within the distance that the air pollution from flaring has been shown to travel. Another important point to note is that flaring in Nigeria typically occurs through elevated vertical stacks, though ground-level pit flares and horizontal flares, particularly at older facilities built in the 1960s and 1970s with lower environmental standards [42]. The empirical measurements at Nigerian flow stations have documented stack heights as low as approximately 8 m [43], while other flare stacks can reach several stories in height [44]. The height at which flaring takes place has important implications for human exposure: elevated stacks disperse combustion by-products over a wider area through atmospheric mixing, with the spatial extent of ground-level pollution depending on stack geometry, emission rates, and prevailing meteorological conditions [1]. Ground-level flaring, by contrast, concentrates pollutants closer to the breathing zone, increasing local exposure to harmful substances [45]. The presence of both elevated and ground-level flaring across the Niger Delta means that nearby communities may face varying levels of exposure depending on the infrastructure characteristics of individual flare sites. The use of our inverse-distance weighted measure of flaring exposure captures the aggregate burden from all flare sites regardless of stack configuration, though it does not distinguish between these different modes of flaring.

We have three groups of outcome variables. First, we examine the reported incidence of diseases in young children such as fever, cough, and respiratory issue (this is defined as reporting either one of the following symptoms: cough, blocked nose, short breath, and blocked chest), as we expect our mechanism of impact to be air pollution, which we show in the incidence of these diseases. We include diarrhoea as a placebo test for our analysis, as we expect the air pollution channel not to be associated with diarrhoea. Second, anthropometric measurements yield z-scores for height-for-age (HAZ), weight-for-height (WHZ), and weight-for-age (WAZ). A child is considered stunted, wasted, or underweight if HAZ, WHZ or WAZ for the child is less than two standard deviations below the median measurement for the reference group [46]. Third, measures of mortality at the cluster level, specifically infant mortality (the proportion of children who died before they are 12 months old), child mortality (the proportion of children who died when they were between 12 and 59 months old), and under-5 mortality (the proportion of children under 59 months old who died).

To establish the association between flaring and child health outcomes, we regress the child health outcome variables on risk of exposure to gas flaring controlling for child, parent, and household characteristics. Previous research [4,5] and data availability in our context guided the choices of control variables. We run our regressions at two levels. We take both a child-level approach and an aggregated cluster level approach. Both approaches allow us to examine associations at individual and community level.

Under the cluster-level approach, we aggregate the incidence of diseases (disease rate), health outcomes (probability of stunting, wasting, and being underweight), and child-level mortality to the cluster level and run a regression of these outcomes on the risk of flaring for each cluster with cluster level controls (averages). The main specification under this approach is:

(2) $$Y_{ct}=\alpha +\;\beta_{1}{RF}_{ct}+\sum_{d=1}^{D}\theta_{d}X_{ct}^{d}+\gamma_{t}+\epsilon_{ct}$$

where $Y_{ct}$ are measures of child health, i.e., the incidence of cough, respiratory symptoms, fever, diarrhoea, infant, child, and under-5 mortality in the cluster, c, in year, t. ${RF}_{ct}$ is the flaring exposure for the DHS cluster, c, in time, *t*. The $X_{ct}$ are control variables at the cluster level, $\gamma_{t}$ are year effects, and $\epsilon_{ct}$ are the idiosyncratic errors. The main coefficient of interest, $\beta_{1}$, indicates the association between gas flaring and the rates of child morbidity and mortality at the cluster level.

At the individual level, we run regressions of whether each individual child has a particular disease or condition on exposure to flaring for each cluster while controlling for individual, parental, and household characteristics with clustered standard errors at the DHS cluster level:

(3) $$Y_{ict}=\alpha +\;\beta_{2}{RF}_{ct}+\sum_{d=1}^{D}\theta_{d}X_{ict}^{d}+\gamma_{t}+\epsilon_{ict}\;$$

where $Y_{ict}$ is the outcome variable measures of each child, i, in cluster, c, at time, t, in year, t. ${RF}_{ct}$ is the exposure to gas flaring for the DHS cluster, c, in time, t. $X_{ict}$ are the vectors of individual, parental, and household control variables, $\gamma_{t}$ are the year effects for each DHS-year, and $\epsilon_{ict}$ represents the idiosyncratic errors. The main coefficient of interest, $\beta_{2}$, indicates the association between child health outcomes and flaring at the individual level.

## 3. Results and Discussion

Table 1 has annual estimates of flaring from 2012 to 2018 (covering timing of the DHS), comparing satellite-detected estimates with the official estimates reported by the Department of Petroleum Resources (DPR). The satellite-detected estimates are broken down by location, while the DPR figures are just totals, as it does not further break down flaring by location. We examine both offshore and onshore flaring and aggregate onshore flaring to the state level (the second administrative level). Flaring volumes show that around half of all gas flared happens offshore. About 56% of flaring in 2013 was offshore, reducing to 48% in 2018. With respect to onshore flaring, Delta State has the highest flaring by volume, with around a quarter of all gas flared in 2018 in this state. Two states (Ondo and Anambra) had gas flared in 2013 but not at the time of our second DHS in 2018. The gap between satellite estimates and the official data vary by year. In 2013, the official estimates are 2 percent higher than satellite estimates, while in 2018, the official estimates are around 32 percent lower than the satellite estimates. From 2015 onward, official estimates are lower than satellite estimates. One possible explanation is increased under-reporting by companies in anticipation of stricter flaring penalties, although differences in reporting and measurement practices may also contribute (for example, from 2017, the government had approved the National Gas Policy and the Gas Flaring Prohibition Bill was deliberated upon by the Senate in March 2017, with public hearing for stakeholders in the industry and the public in May 2017). The under-reporting of the official estimates is also consistent with the evidence from other settings, which show that self-reported data often misrepresent actual emissions [47]. An example from the Permian basin in the US reported by the Environmental Defense Fund indicates that operators reported around half of the volume of gas flared compared to the satellite data [48]. Significant discrepancies between the industry-reported flaring data and satellite data are also reported in Texas, North Dakota, and New Mexico [49]. Thus, it is not surprising to see patterns of under-reporting in Nigeria, particularly in the context of regulatory changes that increase the penalty for flaring by around 80-fold.

**Table 1:** Gas volume flared in million standard cubic feet (MMSCF) by location in Nigeria.

Flare Location by Satellite	2012	2013	2014	2015	2016	2017	2018	2013 Share	2018 Share
Abia	1027	2255	2601	1032	1096	1604	1556	1%	0%
Akwa Ibom	3715	6070	4992	5206	5059	5528	5350	1%	1%
Anambra	91	610	533	414	-	-	-	0%	0%
Bayelsa	39,400	38,600	45,100	32,500	42,000	42,600	50,000	9%	11%
Delta	46,900	59,000	54,500	59,600	45,500	66,800	107,000	14%	23%
Edo	12,200	18,100	18,200	18,800	7324	13,800	23,200	4%	5%
Imo	15,100	14,200	8608	6901	7961	6816	6053	3%	1%
Ondo	2434	2635	2447	-	-	-	-	1%	0%
Rivers	45,600	42,500	47,900	41,800	53,400	53,300	52,200	10%	11%
Offshore	194,000	236,000	204,000	181,000	181,000	182,000	226,000	56%	48%
Total flaring by satellite estimates	480,623	419,970	388,880	347,254	343,340	372,448	471,359	100%	100%
Official flaring estimates from the DPR	465,257	427,971	393,840	330,933	288,917	324,192	321,290		
Gap (as % of satellite total)	3%	2%	1%	−5%	−16%	−13%	−32%		

Note: Table 1 presents the gas flare volume estimates from the satellite data for the Niger Delta States for the period of 2012–2018. The official total flare estimates (self-reported by oil and gas companies) are presented in the second-to-last row. The official estimates are from the Department of Petroleum Resources 2018 Annual report. The report comes with a disclaimer which states that figures presented are subject to ongoing review based on continuous reconciliation with various stakeholders. The official estimates were 1–3% higher than satellite estimates in the period of 2012–2014, but from 2015, the official estimates were less than satellite estimates, and by 2018, the official estimates were around a third lower than satellite estimates. The satellite estimates for 2012 are from the month of March. Annualising the estimates from March leads to a full-year estimate of 480,623 MMSCF. The under-reporting in official estimates from 2015 may reflect under-reporting by gas companies in anticipation of regulatory change that increased the penalty for flaring by 80-fold. Figure 1 identifies all flaring spots, as well as the location of all DHS clusters in the Niger Delta region.

**Figure 1:**
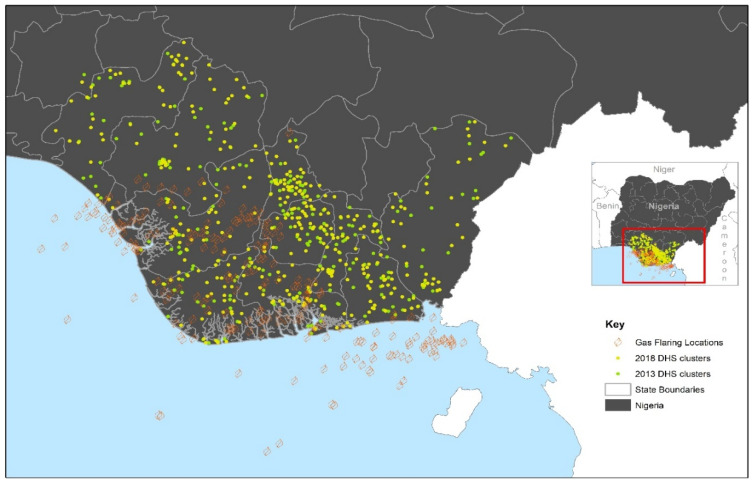
Gas flaring locations (onshore and offshore) and DHS clusters (2013 and 2018) in the Niger Delta region of Nigeria. Note: Figure 1 indicates the proximity of flare sites by presenting the location of gas flares and 2013 and 2018 DHS clusters.

The median distance between the DHS clusters and each flaring site is 181 km (in both 2013 and 2018), including onshore and offshore flaring sites. For onshore sites, the median distance is 153 km. The closest DHS cluster to a flare site in 2018 (2013) was 0.87 km (0.69 km).

Table 2 presents the summary statistics from the pooled 2013 and 2018 Demographic Health Surveys for our child health outcomes, parental, and household characteristics in the oil-producing states (including Cross River State).

Table 3 shows the associations between flaring, the incidence of disease, and nutritional outcomes at the individual level, controlling for parental and household characteristics. We find a positive association between the flaring exposure and the incidence of all the diseases considered, except diarrhoea. A standard deviation higher flare volume is associated with a three-percentage point higher probability that a child has cough, a respiratory symptom, or fever. The positive association between flaring and incidence of respiratory diseases and fever are consistent with air pollution as a channel driving the association. Fever is often a symptom of many respiratory diseases, including acute respiratory infections, colds, pneumonia, and sore throats [50,51]. The lack of association between diarrhoea and flaring is consistent with air pollution as one possible channel driving the association between flaring and incidence of diseases. Our results are also in line with the evidence from [26], where the analysis of medical records showed a greater frequency of disease types such as asthma, cough, breathing difficulty, and eye/skin irritation in areas with a long history of gas flaring compared to areas with no flaring. Among the control variables, child age, the education of the mother’s partner, and persons per room (an indicator of crowding) had a significant association with flaring. The coefficients for wealth are mostly negative across disease incidence, and anthropometric outcomes suggesting that children from wealthier households tend to have somewhat better health outcomes. These associations are generally not statistically significant, except for diarrhoea, where children in the middle and richest wealth quintiles have significantly lower incidence. The lack of a strong, monotonic gradient across wealth quintiles may reflect the relatively limited variation in socioeconomic status within the Niger Delta sample, where even households classified as wealthier by the national DHS standards may still face substantial environmental exposures, given the widespread nature of flaring in this region.

For child anthropometric characteristics, we find significant positive associations between flaring and the probability that a child is underweight or wasted but no association with stunting. Weight-based child anthropometric indicators are typically considered to reflect shorter-term nutritional deprivation and morbidity, while stunting reflects longer-term conditions. It is also important to distinguish between the mechanisms through which flaring may affect different outcome categories. The positive associations between flaring and the incidence of cough, respiratory symptoms, and fever are consistent with a direct air pollution pathway, as inhalation of combustion by-products can trigger acute respiratory illness and febrile responses. In contrast, stunting, wasting, and underweight are primarily indicators of nutritional status and are more strongly determined by dietary adequacy, feeding practices, and the disease environment broadly. The association between flaring, wasting, and being underweight (both shorter-term nutritional indicators) may operate through indirect channels: repeated illness episodes (such as respiratory infections) can reduce appetite and nutrient absorption [52], and household resources may be diverted from nutrition to healthcare. The absence of a significant association between flaring and stunting, a longer-term cumulative measure of nutritional deprivation, is consistent with this interpretation, as our exposure measure captures relatively recent flaring and may not reflect the sustained deprivation that drives chronic growth faltering.

**Figure A1:**
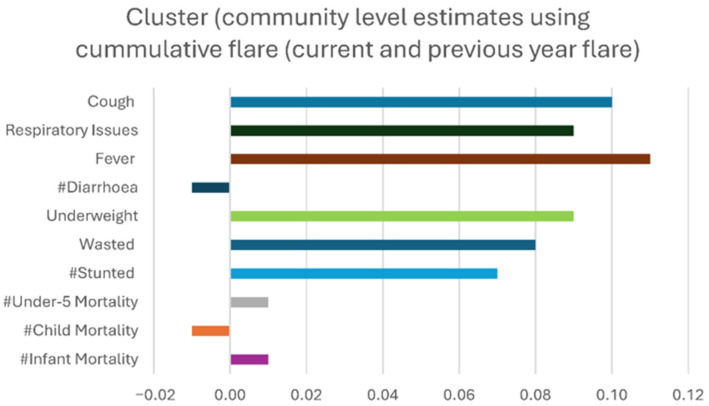
Estimates of cluster (community)-level association between cumulative exposure to gas flares (current and cumulative) and child health; nutritional and mortality outcomes, with controls for cluster level characteristics. Note: The cluster-level results use as control variables: the cluster averages for wealth quintile and the average person per room, the proportion of households in the cluster with poor wall and poor roof, and the proportion of mothers who read a newspaper (as a proxy for access to information). We find statistically significant positive associations between an increase in flaring and incidence of respiratory disease, cough, and fever but no association with diarrhoea. For nutritional outcomes, we find positive associations between an increase in flaring and short-term nutritional outcome measures. For the cumulative analysis, we find no relationship with stunting nor with child mortality outcomes. # is in front of outcomes that are not statistically significant.

We include the effect of flaring in prior years on child health and nutritional outcomes by examining the cumulative effect of flaring in the current year and in the previous year (reported in Appendix A Figure A1). The availability of the flaring data limits us to examining the effect of previous historical flaring. The flaring data is only available since 2012. To ensure that we use information from both 2013 and 2018 DHS and not to lose most of our sample, we define our cumulative exposure as flaring in the current period and in the previous year. Using cumulative exposure (see Appendix A Figure A1), we find statistically significant positive association between flaring and incidences of cough, respiratory diseases, and fever. For nutritional outcomes, we find that flaring is positively associated with being wasted and underweight. Compared to estimates using current year flares only, the magnitude of the estimates using cumulative exposure is greater for disease incidences but smaller for nutritional outcomes, and we find no association between flaring and stunting. Our analysis using cumulative exposure reinforces our previous findings on the association between flaring and the incidence of diseases and indicates an association between flaring and shorter-term nutritional outcomes, although we cannot account for previous historical flaring beyond the previous year due to the satellite data beginning from 2012 (the satellite data on flaring is available from 2012; for the cumulative exposure analysis, we focus on flares in the current year and the year before, which allows us to include both the 2013 and 2018 DHS samples).

**Figure 2:**
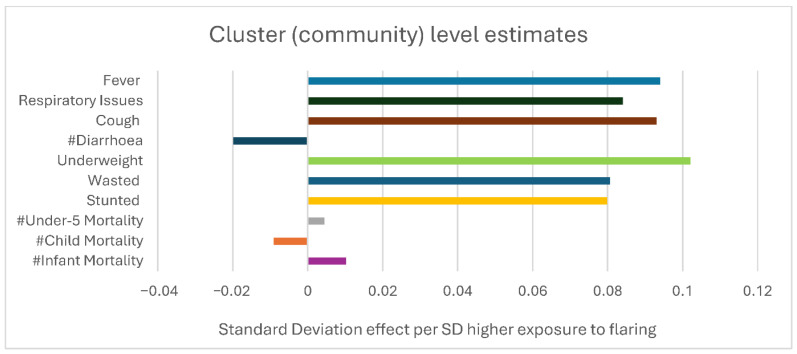
The estimates of cluster (community)-level association between gas flaring and child health, as well as nutritional and mortality outcomes with controls for cluster level characteristics. Note: cluster (community)-level association between gas flaring and child health, as well as nutritional and mortality outcomes controls for cluster level characteristics. The cluster-level results use as control variables the cluster averages for wealth quintile and the average person per room, proportion of households in the cluster with poor wall and poor roof, and the proportion of mothers who read newspapers (as a proxy for access to information). We find statistically significant positive associations between an increase in flaring and incidence of respiratory disease, cough, and fever but no association with diarrhoea. For nutritional outcomes, we find positive associations between an increase in flaring and all nutritional outcome measures but no relationship with mortality. # is in front of outcomes that are not statistically significant.

Figure 2 shows the associations between the flaring exposure and the incidence of disease, nutritional outcomes, and mortality rates at the cluster (community)-level, controlling for parental and household characteristics (full results in Appendix A Table A2). The cluster level results provide estimates of associations at the community level. We include the cluster averages for wealth quintile, the average person per room, proportion of households in the cluster with poor wall and poor roof, and the proportion of mothers who read newspapers (as a proxy for access to information). We find a positive association between flaring exposure and incidence rates of all diseases considered, except diarrhoea. The results for respiratory health imply that one SD increase in the volume of gas flared raises the cluster-level cough rates by 0.093 SD; other respiratory issues rate by 0.084 SD and fever rates by 0.094 SD. Given the standard deviation of these diseases across all clusters, the results suggest that a standard deviation increase in flaring is associated with an increase in the reported cough rate that is equivalent to 22% of the mean cough incidence across all clusters in both periods. The results for other diseases indicate that a standard deviation increase in flaring is associated with an increase in respiratory issues that are around 18 percent of mean incidence of respiratory issues and around 14 percent of the mean incidence of fever across all clusters in both periods. We find positive associations between flaring and both short-term and long-term measures of nutritional outcomes at the community level.

It is important to note that our study does not capture the full range of health symptoms that have been documented in communities exposed to oil and gas production. Research from fracking sites in developed countries has identified a broader set of non-specific symptoms, including headaches, fatigue, cognitive impairment, and nosebleeds [53,54]. In the Nigerian context, community-level surveys have reported similar complaints among residents of flaring host communities, including headaches, skin irritation, and eye problems, alongside the respiratory symptoms that are the focus of this study [26]. However, the DHS does not collect data on these non-specific symptoms, limiting our ability to examine this broader symptom profile. Dedicated health surveys in the Niger Delta that capture a wider range of symptoms, including neurological complaints and sensory irritation, would be valuable for building a more complete picture of the health burden associated with gas flaring in this region.

This study has several notable strengths. First, it provides the first large-scale examination of gas flaring and child health in Nigeria, overcoming the data limitations that have previously restricted research to small community-based comparisons. Second, the use of satellite-detected flaring data from the VIIRS provides an objective, externally validated measure of flaring activity that is independent of potentially unreliable self-reported industry data, as our comparison with the official estimates demonstrates. Third, the inverse-distance weighted market potential approach to measuring flaring exposure is well-suited to this context: it accommodates the spatial displacement built into DHS cluster coordinates, weights exposure by flare volume and proximity, and incorporates offshore flaring, which accounts for roughly half of all flaring in Nigeria and which has been overlooked in prior studies. Fourth, the inclusion of both individual-level and cluster-level analyses provides a comprehensive view of associations at both the child and community levels.

Its limitations should also be acknowledged. First, our study design is cross-sectional and observational, so the associations reported here cannot be interpreted as causal effects. Omitted variable bias remains a concern, as unobserved factors correlated with both flaring proximity and child health, such as other forms of oil-related pollution (e.g., oil spills, pipeline leaks), local economic conditions, or healthcare access, may confound our estimates. Second, the child morbidity measures from the DHS are based on maternal recall over a 2-week window, which introduces the possibility of recall bias and measurement error, though this is a well-known feature of the DHS data and is unlikely to be systematically correlated with flaring exposure. Third, we lack direct measurements of air quality or individual-level pollution exposure; our flaring exposure measure is an area-based proxy that does not account for variations in atmospheric conditions, wind direction, stack height, or time spent outdoors.

Another important pollution channel associated with gas flaring is methane. The leaks of methane, often associated with improperly combusted gas during the flaring process, are an important channel through which flaring may affect human health. However, satellite data on methane available over our period of interest are at a regional scale (100–1000 km) and are derived through multi-year averaging [55]. The existing evidence on methane emissions from Nigeria, such as [56], relies on such data for an 0.5 × 0.5-degree grid, which is approximately 3000 sq km at Nigeria’s latitude. This is far too coarse for our purposes. Moreover, they have been aggregated over multiple years. However, future work may be able to account for the role of methane, as the TROPOMI instrument launched in 2017, which can detect large point sources of methane, has started producing data from July 2018.

We also note that it would be useful to include additional controls on oil and gas production, such as total production in geographic clusters and nearby well activity. However, this type of detailed information on oil and gas production is not available or remains very sensitive in settings like Nigeria.

Relatedly, an important caveat to associations between flaring and child health is that, even with the inclusion of information on methane and data on well production, omitted variable bias may still be a problem. In future work, we will investigate the possibility of identifying a causal impact of pollution on health outcomes by comparing the health outcomes of siblings born to the same mother before and after flaring. Sometimes, technical factors cause wells to stop flaring for a long period of time before resuming, and this gives an opportunity to compare the outcomes of siblings born to the same mother before and after flaring for locations with long stoppages in flaring. By comparing siblings born to the same mother, we can control for a host of other unobservable characteristics that may confound the identification of the causal relationship between gas flaring and child health.

## 4. Conclusions

We use a newly available dataset on satellite-detected gas flaring to examine the relationship between flaring and various child health outcomes. Our results show a positive association between flaring and the incidence of diseases (particularly cough, respiratory symptoms, and fever) and short-term nutritional outcomes (wasting and underweight). Our results are in line with earlier evidence from a smaller subset of flaring communities where flaring has been found to be associated with a host of diseases and poor agricultural production. Our analysis provides the first comprehensive overview of flaring and child health outcomes in the Niger Delta region, one of the regions with the highest flare activity in the world and where earlier efforts to examine the impact of flaring activity were limited due to a lack of data. While the methods described above can be used to show an association between flaring and human health, omitted variable bias may still be a problem and the association presented by these methods does not necessarily imply causation. The method and design described for this project can be extended to other countries in the sub-Saharan Africa such as the Republic of Congo and Gabon that are like Nigeria in their practice of routine gas flaring but where unavailability of data has limited the examination of the health impacts of this practice. The extension of the approach described here provides an opportunity to build an evidence base on the impact of gas flaring on human health in the sub-Saharan Africa.

## Figures and Tables

**Table 2:** Summary statistics of child health outcomes and parental and household control variables from pooled 2018 and 2013 DHSs.

Variable	Weighted Obs.	Mean	Std. Dev.	Min	Max
Child Incidence of Disease					
Cough dummy (0/1)	9722	0.17	0.38	0	1
Respiratory issues dummy (0/1)	9789	0.18	0.38	0	1
Fever dummy (0/1)	9720	0.19	0.39	0	1
Diarrhoea dummy (0/1)	9714	0.06	0.23	0	1
Child Anthropometric Characteristics					
Stunted (1 if <−2 SD)	6317	0.18	0.39	0	1
Wasted (1 if <−2 SD)	6316	0.09	0.29	0	1
Underweight (1 if <−2 SD)	6323	0.11	0.32	0	1
Child Mortality					
Infant mortality (under 12 months) rate	10,555	0.05	0.22	0	1
Child mortality (12–59 months) rate	10,555	0.01	0.12	0	1
Under-5 mortality (0–59 months) rate	10,555	0.07	0.25	0	1
Child Characteristics					
Gender (boy child dummy)	9789	0.51	0.50	0	1
Age Of Child (years)	9789	2.37	1.49	0	5
Parental Characteristics					
Mother’s education (years)	9788	9.97	3.94	0	20
Age of Mother at first birth (years)	9789	21.85	4.79	12	48
Mother’s current partner’s education (years)	9006	10.61	3.83	0	20
Mother reads newspaper dummy (0/1)	9771	0.27	0.44	0	1
Household Characteristics					
Has TV Dummy (0/1)	9562	0.73	0.44	0	1
Poorest wealth quintile	9789	0.02	0.13	0	1
Poorer wealth quintile	9789	0.09	0.29	0	1
Middle wealth quintile	9789	0.23	0.42	0	1
Richer wealth quintile	9789	0.32	0.47	0	1
Richest wealth quintile	9789	0.34	0.47	0	1
Fuel type: electricity/LPG/natural gas/biogas)	9565	0.09	0.29	0	1
Fuel type: kerosene	9565	0.33	0.47	0	1
Fuel type: (coal/charcoal/wood/straw/shrubs)	9565	0.58	0.49	0	1
Poor roof dummy (0/1)	9561	0.06	0.23	0	1
Poor wall dummy (0/1)	9562	0.18	0.38	0	1
Person per room (persons/room)	9779	3.34	1.66	0.02	12
Urban or rural (1 = urban/2 = rural)	9789	1.52	0.50	1	2

Note: Table 2 presents the summary statistics of the child health and nutrition outcomes (incidence of disease, anthropometric characteristics, and mortality), as well as parental and household characteristics. The reported wealth quintiles are based on the national wealth distribution.

**Table 3:** Regression of gas flaring on incidence of disease and anthropometric characteristics controlling for individual and household characteristics: individual level.

	(1)	(2)	(3)	(4)	(5)	(6)	(7)
Variables	Child Has Cough	Child Has Respiratory Issues Symptoms	Child Has Fever	Child Has Diarrhoea	Child Is Stunted	Child Is Wasted	Child Is Underweight
Flaring exposure	0.03 ***	0.03 ***	0.03 ***	0.00	0.01	0.01 **	0.01 *
	[0.01]	[0.01]	[0.01]	[0.00]	[0.01]	[0.00]	[0.00]
Boy child	0.01	0.01	−0.00	0.00	0.02 *	0.01	0.02 **
	[0.01]	[0.01]	[0.01]	[0.01]	[0.01]	[0.01]	[0.01]
Mother education (years)	0.00	0.00	0.00 **	0.00	−0.00	−0.00	−0.00 *
	[0.00]	[0.00]	[0.00]	[0.00]	[0.00]	[0.00]	[0.00]
1. Age (years)	0.11 ***	0.11 ***	0.11 ***	0.04 ***	0.00	0.03 *	0.01
	[0.02]	[0.02]	[0.02]	[0.01]	[0.02]	[0.02]	[0.02]
2. Age (years)	0.07 ***	0.06 ***	0.10 ***	0.01	0.10 ***	−0.04 *	0.01
	[0.02]	[0.02]	[0.02]	[0.01]	[0.02]	[0.02]	[0.02]
3. Age (years)	0.06 ***	0.05 ***	0.06 ***	−0.01	0.08 ***	−0.09 ***	−0.02
	[0.02]	[0.02]	[0.02]	[0.01]	[0.02]	[0.02]	[0.02]
4. Age (years)	0.01	0.00	0.04 ***	−0.03 ***	0.04 *	−0.08 ***	−0.01
	[0.02]	[0.02]	[0.01]	[0.01]	[0.02]	[0.02]	[0.02]
5. Age (years)	−0.01	−0.01	0.04 **	−0.02	0.04 *	−0.08 ***	0.00
	[0.02]	[0.02]	[0.02]	[0.01]	[0.03]	[0.02]	[0.02]
Age of Mother at first birth (years)	−0.00	−0.00	−0.00	−0.00	−0.00	0.00	0.00
	[0.00]	[0.00]	[0.00]	[0.00]	[0.00]	[0.00]	[0.00]
Mother’s partner’s education (years)	0.00 **	0.00 ***	0.00	−0.00	−0.00 **	−0.00	−0.00 *
	[0.00]	[0.00]	[0.00]	[0.00]	[0.00]	[0.00]	[0.00]
Has TV	−0.02	−0.02	0.00	−0.01	0.03	−0.01	0.01
	[0.02]	[0.02]	[0.02]	[0.01]	[0.02]	[0.01]	[0.01]
Poorer wealth quintile	−0.01	−0.02	−0.00	−0.07 **	−0.04	0.00	0.03
	[0.04]	[0.04]	[0.05]	[0.04]	[0.06]	[0.03]	[0.04]
Middle wealth quintile	−0.02	−0.03	−0.03	−0.08 **	−0.02	0.00	−0.00
	[0.04]	[0.04]	[0.06]	[0.04]	[0.06]	[0.03]	[0.04]
Richer wealth quintile	−0.02	−0.03	−0.04	−0.06	−0.10	−0.02	−0.06
	[0.04]	[0.04]	[0.06]	[0.04]	[0.06]	[0.03]	[0.04]
Richest wealth quintile	−0.05	−0.06	−0.08	−0.09 **	−0.10	−0.03	−0.06
	[0.05]	[0.05]	[0.06]	[0.04]	[0.06]	[0.03]	[0.04]
Kerosene cooking fuel type	−0.05	−0.05	−0.00	−0.01	0.01	0.01	0.02
	[0.03]	[0.03]	[0.03]	[0.01]	[0.03]	[0.02]	[0.02]
Coal/charcoal/wood/straw shrubs cooking fuel type	−0.05	−0.04	0.01	0.01	0.04	−0.00	0.03
	[0.03]	[0.03]	[0.03]	[0.01]	[0.03]	[0.02]	[0.02]
Poor roof	0.00	0.00	−0.00	0.01	0.02	−0.01	0.01
	[0.03]	[0.03]	[0.04]	[0.02]	[0.04]	[0.02]	[0.03]
Poor wall	−0.00	−0.00	0.01	0.00	0.04 *	−0.02	0.00
	[0.02]	[0.02]	[0.02]	[0.01]	[0.02]	[0.02]	[0.02]
Person per room	0.01 **	0.01 **	0.00	0.00	0.00	0.00	0.00
	[0.00]	[0.00]	[0.00]	[0.00]	[0.00]	[0.00]	[0.00]
Mother reads newspapers	0.01	0.01	0.01	−0.01	−0.02	0.01	−0.01
	[0.01]	[0.01]	[0.01]	[0.01]	[0.02]	[0.01]	[0.01]
Rural	−0.01	−0.02	−0.00	−0.00	−0.01	−0.00	−0.02
	[0.02]	[0.02]	[0.02]	[0.01]	[0.02]	[0.01]	[0.01]
2018. year	0.05 ***	0.06 ***	0.06 ***	0.00	0.03 *	−0.07 ***	−0.01
	[0.02]	[0.02]	[0.02]	[0.01]	[0.01]	[0.01]	[0.01]
Constant	0.10	0.11 *	0.09	0.13 ***	0.23 ***	0.19 ***	0.16 ***
	[0.06]	[0.06]	[0.08]	[0.05]	[0.08]	[0.05]	[0.06]
Observations	9754	9803	9750	9754	6521	6523	6528
R-squared	0.03	0.04	0.03	0.02	0.04	0.04	0.02

Note: Standard errors in brackets *** *p* < 0.01, ** *p* < 0.05, * *p* < 0.1. Regression of gas flaring on incidence of disease and anthropometric characteristics controlling for individual, parental, and household characteristics. Control variables for individual characteristics include the gender and age of the child. Parental characteristics include information pertaining to mother’s age, mother’s age at first child, mother’s education, and whether mother reads newspapers. Household characteristics include information on whether the household has a TV, household wealth quintile, household cooking fuel type, person per room, and whether household has poor roof and poor walls, as well as location (urban or rural). It also includes a dummy for the survey year. We find positive association between flaring and the incidence of cough, respiratory symptoms, and fever. We find no association with diarrhoea. On anthropometric characteristics, we find associations between flaring and wasting and being underweight (measures of short-term nutritional outcomes). Among the control variables, child age, the education level of the mother’s partner, and persons per room (an indicator of crowding) had a significant association with flaring.

## Data Availability

The satellite data on gas flaring is publicly available at www.gasflaretracker.ng (accessed on 1 February 2021). The Demographic Health Surveys data are available upon registration at https://dhsprogram.com (accessed on 1 February 2021).

## Appendix A

**Table A1:** The summary of datasets used.

Dataset	Source	Coverage	Period	Key Variables
VIIRS Nightfire Gas Flaring	NOAA/Suomi satellite via gasflaretracker.ng	All onshore and offshore flare sites in the Niger Delta, Nigeria	2012–2018 (monthly)	Geographic coordinates of flare sites; monthly estimates of flare volume (MMCF)
Nigerian DHS (Round 6)	DHS Programme/NPC	Nationally representative; analysis restricted to the Niger Delta states + the Cross River	2013	Child morbidity (cough, respiratory symptoms, fever, and diarrhoea); anthropometrics (HAZ, WHZ, and WAZ); birth history and mortality; parental and household characteristics; cluster GPS coordinates (displaced up to 2 km urban and up to 10 km rural)
Nigerian DHS (Round 7)	DHS Programme/NPC	Nationally representative; analysis restricted to the Niger Delta states	2018	Same as DHS 2013 above

**Table A2:** Regression of incidence of disease and mortality on gas flaring at the cluster (community level) controlling for cluster characteristics.

	(1)	(2)	(3)	(4)	(5)	(6)	(7)	(8)	(9)	(10)
Variables	Cough Rate	Respiratory Issue Rate	Fever Rate	Diarrhoea Rate	Stunting Rate	Wasting Rate	Underweight Rate	Infant Mortality Rate	Child Mortality Rate	Under-5 Mortality Rate
Flaring exposure	0.09 **	0.08 **	0.09 **	−0.02	0.08 **	0.08 *	0.10 **	0.01	−0.01	0.00
	[0.04]	[0.04]	[0.04]	[0.04]	[0.04]	[0.04]	[0.04]	[0.04]	[0.04]	[0.04]
Wealth quintile	−0.07	−0.07	−0.22 ***	−0.17 **	−0.28 ***	−0.11	−0.27 ***	−0.14 **	−0.14 **	−0.18 ***
	[0.07]	[0.07]	[0.07]	[0.07]	[0.07]	[0.07]	[0.07]	[0.07]	[0.07]	[0.07]
Poor roof rate	−0.02	−0.02	0.01	0.02	−0.02	−0.01	0.05	−0.06	0.02	−0.05
	[0.05]	[0.05]	[0.05]	[0.05]	[0.05]	[0.05]	[0.05]	[0.05]	[0.05]	[0.05]
Poor wall rate	−0.04	−0.06	−0.14 **	−0.14 **	0.01	−0.09	−0.09	−0.03	−0.06	−0.05
	[0.06]	[0.06]	[0.06]	[0.06]	[0.06]	[0.06]	[0.06]	[0.06]	[0.06]	[0.06]
Persons per room	0.10 **	0.10 **	0.04	0.01	0.05	0.01	0.04	−0.00	−0.01	−0.00
	[0.04]	[0.04]	[0.04]	[0.04]	[0.04]	[0.04]	[0.04]	[0.04]	[0.04]	[0.04]
Reads newspaper rate	0.06	0.05	0.07	−0.04	−0.11 **	−0.01	−0.07	0.06	−0.00	0.05
	[0.05]	[0.05]	[0.05]	[0.05]	[0.05]	[0.05]	[0.05]	[0.05]	[0.05]	[0.05]
Rural	0.02	−0.01	0.04	0.01	−0.03	0.00	−0.07	0.23 **	−0.02	0.20 **
	[0.10]	[0.10]	[0.10]	[0.10]	[0.09]	[0.10]	[0.09]	[0.10]	[0.10]	[0.09]
Year = 2018	0.00	−0.00	0.00	0.00	−0.00	−0.00	−0.00	0.00	−0.00	0.00
	[0.08]	[0.08]	[0.08]	[0.08]	[0.08]	[0.08]	[0.08]	[0.08]	[0.08]	[0.08]
Constant	−0.01	0.01	−0.02	−0.00	0.02	−0.00	0.04	−0.14	0.01	−0.12
	[0.09]	[0.09]	[0.09]	[0.09]	[0.08]	[0.09]	[0.09]	[0.09]	[0.09]	[0.09]
Observations	603	603	603	603	595	595	595	603	603	603
R-squared	0.02	0.02	0.03	0.02	0.12	0.01	0.08	0.03	0.01	0.03

Note: Standard errors in brackets *** *p* < 0.01, ** *p* < 0.05, * *p* < 0.1. Cluster (community)-level association between gas flaring and child health; nutritional and mortality outcomes controls for cluster-level characteristics. The cluster-level results use as control variables the cluster averages for wealth quintile and the average person per room, the proportion of households in the cluster with poor wall and poor roof, and the proportion of mothers who read a newspaper (as a proxy for access to information). We find statistically significant positive associations between an increase in flaring and incidence of respiratory disease, cough, and fever but no association with diarrhoea. For nutritional outcomes, we find positive associations between an increase in flaring and all nutritional outcome measures but no relationship with mortality.
